# Development and Clinical Deployment of the Systematic Urgent Team Response in the Operating Room (SUTO) Protocol for Intraoperative Massive Hemorrhage: A Technical Report

**DOI:** 10.7759/cureus.111648

**Published:** 2026-06-28

**Authors:** Misako Suto, Takehiko Hanaki, Naoki Moriyama, Akihiro Otsuki, Yoshiyuki Fujiwara

**Affiliations:** 1 Division of Operating Room, Tottori University Hospital, Yonago, JPN; 2 Division of Medical Education, Tottori University, Faculty of Medicine, Yonago, JPN; 3 Division of Gastrointestinal and Pediatric Surgery, Tottori University, Faculty of Medicine, Yonago, JPN; 4 Division of Anesthesiology and Critical Care Medicine, Tottori University, Faculty of Medicine, Yonago, JPN

**Keywords:** crisis resource management, emergency response algorithm, intraoperative bleeding, massive hemorrhage, multidisciplinary team, operating room crisis, patient safety, simulation-based training, suto protocol

## Abstract

Massive intraoperative hemorrhage is a time-critical event requiring simultaneous hemodynamic resuscitation, bleeding source control, transfusion support, equipment preparation, and escalation to multidisciplinary support. This technical report provides, to our knowledge, the first English-language description of the Systematic Urgent Team Response in the Operating Room (SUTO) protocol for intraoperative massive hemorrhage. The protocol was previously developed and reported in Japanese as an institution-specific response system. This English-language report was prepared with permission from the original journal and describes the protocol for an international readership, with additional emphasis on simulation-based preparation and clinical deployment during an actual episode of intraoperative massive hemorrhage. The SUTO protocol was designed to convert the general principles of hemorrhage management into a locally executable, role-based workflow, with predefined procedures for emergency activation, command assignment, task allocation, transfusion support, equipment mobilization, and escalation to cardiovascular surgery and interventional radiology support. The protocol was posted at the entrance of each operating room as an environmental cognitive aid and linked to a dedicated massive hemorrhage cart. Multidisciplinary in situ simulation was conducted using actual operating room equipment to rehearse emergency activation, parallel task execution, transfusion preparation, and escalation pathways. Clinical deployment is illustrated by an unexpected massive hemorrhage caused by splenic artery injury during surgery for esophagogastric junction cancer. Emergency activation, commander declaration, transfusion preparation, equipment mobilization, and consultation with cardiovascular, hepatopancreatobiliary, and interventional radiology teams proceeded in parallel. Definitive hemostasis was achieved by proximal and distal ligation of the injured splenic artery, and the patient was discharged on postoperative day 17. Although causality cannot be inferred from a single clinical deployment, this report suggests that the SUTO protocol may provide a shared operational framework for rapid multidisciplinary coordination during high-acuity operating room crises. Regular simulation and feedback-driven revision are required to maintain organizational readiness as staff and workflows change.

## Introduction

Massive intraoperative hemorrhage is a life-threatening perioperative event that can rapidly transform a controlled operative field into a critical situation [1-4]. Once severe bleeding occurs, the operating room team must simultaneously maintain circulation, control the bleeding source, arrange blood transfusion, secure monitoring and vascular access, prepare specialized equipment, and mobilize additional personnel. Because these actions must occur within minutes, effective management depends on both individual technical expertise and the team's capacity to function as a coordinated system [5,6].

In real clinical settings, however, such an organization can be difficult to maintain during an unexpected crisis. Acute stress, rapidly changing hemodynamics, and incomplete information may fragment communication and obscure task priorities [7,8]. When anesthesiologists, surgeons, nurses, clinical engineers, and other support staff must act in parallel, unclear leadership or role allocation can result in delays, duplicated efforts, or missed tasks. Human factors guidance and crisis resource management literature have therefore emphasized the importance of structured communication, cognitive aids, and clearly defined leadership during critical events [9,10].

Preparedness for massive intraoperative hemorrhage should therefore be established before an emergency occurs. General guidelines, emergency manuals, and crisis checklists provide important principles for managing critical events, and simulation-based training can help staff become familiar with expected roles and workflows [10-12]. However, the practical execution of a massive hemorrhage response is strongly influenced by local conditions, including staffing patterns, blood bank logistics, availability of clinical engineers, access to cardiovascular surgical or interventional radiology support, and the location of specialized equipment. For this reason, general recommendations should be operationalized into practical local protocols that reflect each institution’s resources, communication pathways, role structure, and escalation procedures [13].

In this report, massive intraoperative hemorrhage is understood pragmatically as a time-critical intraoperative event in which ongoing blood loss exceeds the capacity of the current operative team and requires immediate parallel actions, including hemodynamic resuscitation, bleeding source control, transfusion support, equipment preparation, and multidisciplinary escalation. Unlike a conventional massive transfusion protocol, which primarily standardizes blood product delivery, or a general operating room crisis checklist, the SUTO protocol was designed as a locally executable, role-based workflow that specifies emergency activation, command assignment, profession-specific task allocation, equipment mobilization, and escalation pathways. Activation is based on clinical judgment rather than fixed quantitative thresholds, which may permit earlier activation during unexpected or rapidly evolving bleeding; however, this approach may also introduce variability in activation timing depending on staff experience and situational awareness.

At our institution, the Systematic Urgent Team Response in the Operating Room (SUTO) protocol was developed as an institution-specific response system for intraoperative massive hemorrhage and was previously reported in Japanese from the perspective of perioperative nursing and patient safety [14]. To our knowledge, no English-language description of a nurse-coordinated, multidisciplinary protocol for intraoperative massive hemorrhage originating from a Japanese academic institution has been published. The present technical report therefore describes the SUTO protocol for an international readership, with particular emphasis on its multidisciplinary implementation, simulation-based preparation, and clinical deployment during an actual episode of unexpected intraoperative massive hemorrhage.

This article is a translated and adapted English-language version of the Japanese article entitled “Development of an Institution-Specific Massive Hemorrhage Algorithm: Contribution of Perianesthesia Nurses to Patient Safety,” originally published in the Journal of the Japanese Association for Operating Room Technology in 2023 [14]. The present English-language article includes additional explanatory details to clarify the protocol, workflow, and clinical context for an international readership.

## Technical report

Development of the SUTO protocol

The SUTO protocol refers to the English-language presentation of our institution-specific massive hemorrhage response system, the core algorithmic workflow of which was developed in 2019 and previously reported in Japanese [14]. The protocol was designed to standardize multidisciplinary emergency responses during severe intraoperative bleeding by translating general principles of critical hemorrhage management into a locally executable operating room workflow. Its development was informed by domestic guidance on critical hemorrhage management and by local challenges identified through prior experience with intraoperative massive hemorrhage, including delays in equipment preparation, insufficient transfusion information management, lack of shared understanding among professions, and unclear communication pathways [5,14,15].

The initial framework of the SUTO protocol was drafted by a perianesthesia nurse after prior experience with critical intraoperative hemorrhage. It was refined through iterative consultation with anesthesiologists, cardiovascular surgeons, certified perioperative nurses, clinical engineers, and operating room leadership. Rather than being developed through a single formal consensus meeting, practical consensus was built through repeated discussions during routine clinical work regarding feasible communication routes, anticipated hemorrhage-control options, equipment preparation, and contact pathways. The protocol was subsequently approved through the operating room governance process. Because massive intraoperative hemorrhage is encountered infrequently, the SUTO protocol was designed to support an organized system-level response rather than reliance on individual experience alone.

Core components of the SUTO protocol

**Figure 1:**
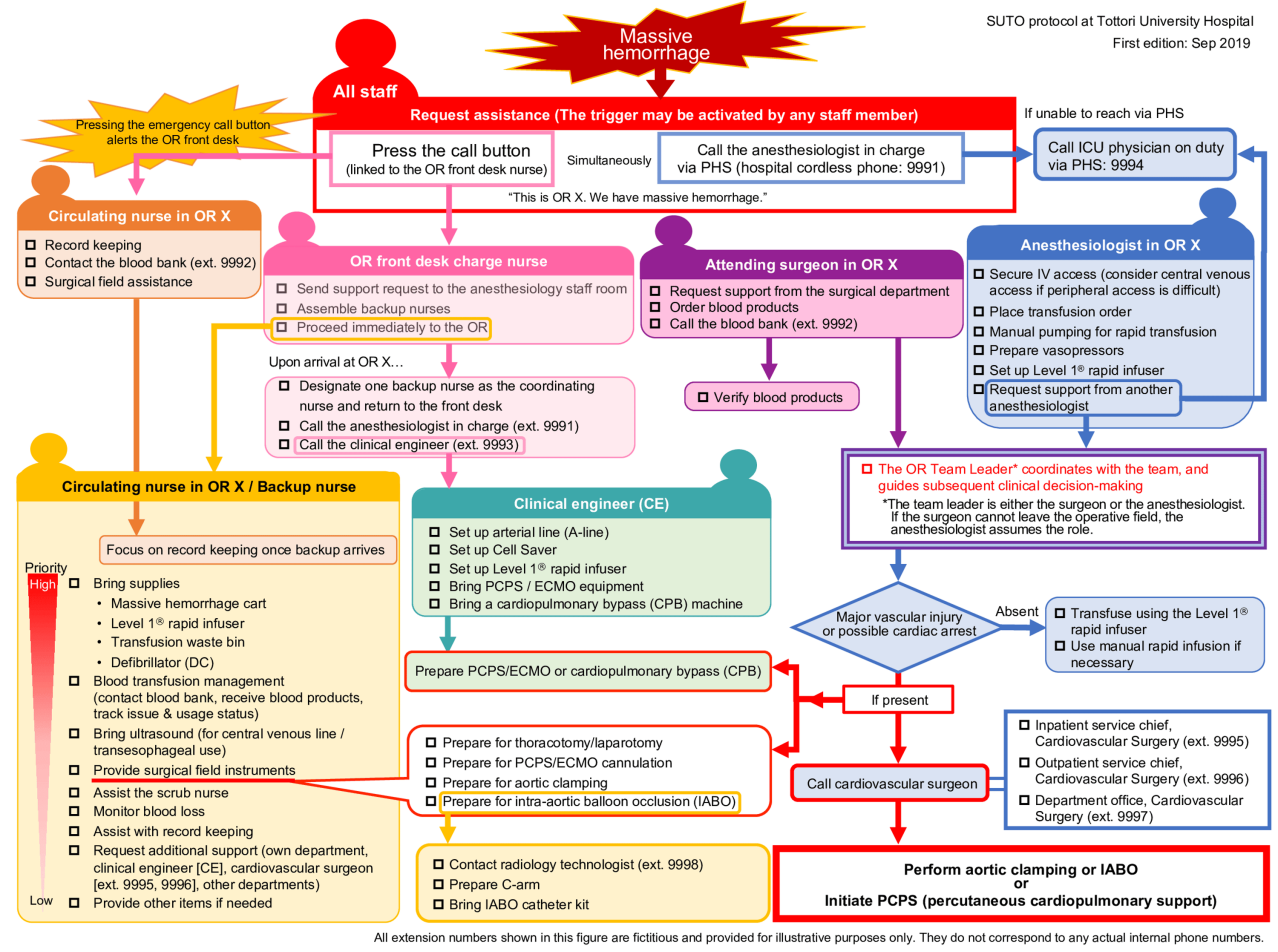
Algorithmic workflow of the SUTO protocol for intraoperative massive hemorrhage. The figure shows the role-based operational workflow of the SUTO protocol, an institution-specific response system used at Tottori University Hospital since 2019. The workflow outlines emergency activation, command assignment, task allocation, transfusion support, equipment mobilization, and escalation to cardiovascular surgical or circulatory support when needed. Colored boxes indicate role groups and professional responsibilities within the workflow. OR X, anonymized operating room number. This figure was translated and adapted by the authors from the original Japanese publication [14] with permission from the publisher and the journal. This figure was created by the authors using Microsoft PowerPoint. No AI-generated images were used. Abbreviations: SUTO, Systematic Urgent Team Response in the Operating Room; OR, operating room; OR X, anonymized operating room; PHS, personal handy-phone system/hospital cordless phone system; ICU, intensive care unit; IV, intravenous; CE, clinical engineer; A-line, arterial line; PCPS, percutaneous cardiopulmonary support; ECMO, extracorporeal membrane oxygenation; CPB, cardiopulmonary bypass; IABO, intra-aortic balloon occlusion; DC, direct-current defibrillator; ext., extension number

As shown in Fig. 1, the algorithmic workflow of the SUTO protocol links emergency activation, parallel task allocation, transfusion support, equipment mobilization, and escalation to advanced hemorrhage-control measures. Its purpose is not only to identify the major tasks required during massive intraoperative hemorrhage but also to specify who initiates them, how support staff is mobilized, and how higher-level interventions are prepared within the operating room system.

The SUTO protocol is organized around four interrelated operational elements. Protocol activation is based on clinical judgment rather than predefined quantitative thresholds for blood loss or hemodynamic parameters. Any staff member who perceives that ongoing hemorrhage exceeds the capacity of the current team, particularly when the clinical course is unexpected, is empowered to initiate the emergency call. The emergency call button activates the operating room front desk, while the responsible anesthesiologist is simultaneously contacted by telephone. The front desk or charge nurse then assembles backup nurses, contacts the anesthesiology staff room and clinical engineers, and dispatches personnel to the operating room. A short scripted emergency message is specified to reduce communication failure during a chaotic situation [9]. Once backup personnel have assembled, command is assigned to either the surgeon or the anesthesiologist; when the surgeon cannot leave the operative field, the anesthesiologist assumes the commander role [9]. Each team member then executes predefined, profession-specific tasks in parallel. The circulating and backup nurses manage record keeping, transfusion, blood loss monitoring, surgical field assistance, and preparation of required supplies, including the massive hemorrhage cart, Level 1 rapid infuser (ICU Medical, San Clemente, CA), transfusion waste container, defibrillator, and ultrasound equipment. The anesthesiologist secures intravenous access, places transfusion orders, prepares vasopressors, and initiates rapid transfusion, while the attending surgeon requests departmental support and coordinates with the blood bank as needed. Clinical engineers prepare the arterial line, Cell Saver, Level 1 rapid infuser, and, when necessary, percutaneous cardiopulmonary support or cardiopulmonary bypass equipment, enabling transfusion support, hemodynamic management, equipment preparation, and operative planning to proceed simultaneously [6,16]. In the event of major vascular injury or possible cardiac arrest, cardiovascular surgeons are contacted, and preparations are initiated for thoracotomy or laparotomy, percutaneous cardiopulmonary support cannulation, cardiopulmonary bypass, aortic clamping, and intra-aortic balloon occlusion [5]. A radiology technologist is also contacted, and the C-arm and intra-aortic balloon occlusion catheter kit are prepared, enabling escalation to advanced circulatory or endovascular support when conventional measures prove insufficient.

Implementation into routine operating room practice

The SUTO protocol was implemented as an environmental cognitive aid within the operating room rather than as a document stored separately from clinical practice. A printed version of the algorithmic workflow was posted at the entrance of each operating room, providing a standardized reference point for staff members who entered the room after emergency activation. This location was selected because additional personnel usually gather near the operating room doorway during a crisis, and a consistent display location across rooms allowed staff to access the same workflow regardless of where the event occurred. The display was positioned so that it could be readily reviewed by staff while avoiding direct visibility to patients entering the operating room.

Equipment preparation was also standardized to support the workflow specified in the SUTO protocol. A dedicated massive hemorrhage cart was reorganized to consolidate supplies required during the early phase of hemorrhage management, including materials for rapid transfusion, emergency laboratory testing, and vascular access support. The cart was arranged so that necessary items could be retrieved according to the expected sequence of actions, reducing repeated movement between the operating room and storage areas. By linking the protocol to a dedicated equipment system, the response was designed to be executable at the bedside rather than dependent on individual memory or ad hoc supply gathering.

This implementation strategy created a shared operational platform for multidisciplinary response. The posted algorithmic workflow clarified the overall response sequence, while the equipment cart supported immediate task execution. Together, these measures were intended to make role allocation, transfusion preparation, equipment mobilization, and escalation pathways visible to all responding personnel. After embedding the SUTO protocol into the operating room environment, education and simulation-based preparation were introduced to ensure that staff could use the system effectively during actual emergencies.

Education and simulation-based preparation

After the SUTO protocol had been embedded into the operating room environment, education and simulation-based preparation were conducted to ensure that staff could use it as an executable workflow during an actual crisis. The educational component consisted of lectures for operating room nurses that explained the rationale behind the protocol, including the pathophysiological basis of hemorrhage management, the importance of early transfusion, and the differences between cardiopulmonary bypass and extracorporeal membrane oxygenation. These sessions were intended not only to introduce the SUTO protocol but also to help staff understand why each action was required during a massive hemorrhage.

Multidisciplinary in situ simulation was then conducted to translate this knowledge into coordinated team performance. The simulation scenario involved an unexpected massive hemorrhage during robot-assisted surgery and included emergency undocking of the robotic system, conversion to open surgery with thoracotomy or laparotomy as required, preparation for surgical or circulatory hemorrhage-control support, and initiation of transfusion. Actual operating room equipment and dummy blood products were used to reproduce the practical workflow as closely as possible.

Before the clinical deployment described in this report, one multidisciplinary in situ simulation session was conducted with 29 participants, including anesthesiologists, thoracic surgeons, cardiovascular surgeons, operating room nurses, clinical engineers, transfusion laboratory technologists, pharmacists, and operating room leadership. A post-training questionnaire assessed perceived usefulness for clinical practice, confidence, satisfaction, and perceived need for continued simulation. However, predefined objective performance endpoints, task completion times, communication error counts, and pre- and post-training comparative measures were not collected. During the simulation, participants practiced emergency activation, role allocation, transfusion preparation, equipment mobilization, and escalation to surgical or circulatory support according to the SUTO protocol. Particular emphasis was placed on shared recognition of the command structure, timely communication of task completion, and parallel execution of profession-specific roles. Informal post-training feedback suggested that the simulation helped participants recognize their roles, understand the overall response workflow, and appreciate the usefulness of the SUTO protocol for clinical practice.

This education and simulation process was designed to convert the posted algorithmic workflow and equipment system into a functional team response. By repeatedly linking the environmental cognitive aid, massive hemorrhage cart, and multidisciplinary workflow, the preparation aimed to reduce hesitation during actual emergencies and allow staff members to enter the crisis response with a shared mental model. The following section illustrates how this prepared system was deployed during an actual episode of unexpected massive intraoperative hemorrhage.

Illustrative clinical deployment

The prepared system was deployed during an unexpected episode of massive intraoperative hemorrhage in a 53-year-old man undergoing surgery for esophagogastric junction cancer. The planned procedure consisted of robot-assisted subtotal esophagectomy with two-field lymph node dissection, retrosternal gastric conduit reconstruction using hand-assisted laparoscopic surgery, and jejunostomy creation. The patient had a history of deep vein thrombosis and current smoking, and his American Society of Anesthesiologists physical status was II.

After completion of the thoracic procedure in the prone position, the patient was repositioned to the supine position, and lymph node dissection along the superior border of the pancreas was performed using hand-assisted laparoscopic surgery. The patient had been hemodynamically stable immediately before the event. During lymph node dissection, however, the splenic artery was injured, resulting in massive hemorrhage. Because splenic artery injury was not initially suspected, identification of the bleeding source was difficult. The patient’s blood pressure decreased rapidly, and 4 minutes after hemorrhage onset, the systolic blood pressure was 39 mmHg, and the heart rate was 132 beats/min. Blood loss reached 1,900 mL within 10 minutes after hemorrhage onset.

In consultation with the attending anesthesiologist, the circulating nurse activated the emergency call according to the SUTO protocol two minutes after hemorrhage onset. A backup nurse arrived 40 seconds after the call, followed immediately by an additional anesthesiologist, and seven personnel assembled within 1 minute of emergency activation. After arrival, the backup nurse first confirmed the need for a transfusion. The senior anesthesiologist then promptly ordered blood products and explicitly declared that he would serve as the commander. This declaration centralized information regarding blood loss, transfusion preparation, and consultation with other departments to the commander.

**Figure 2:**
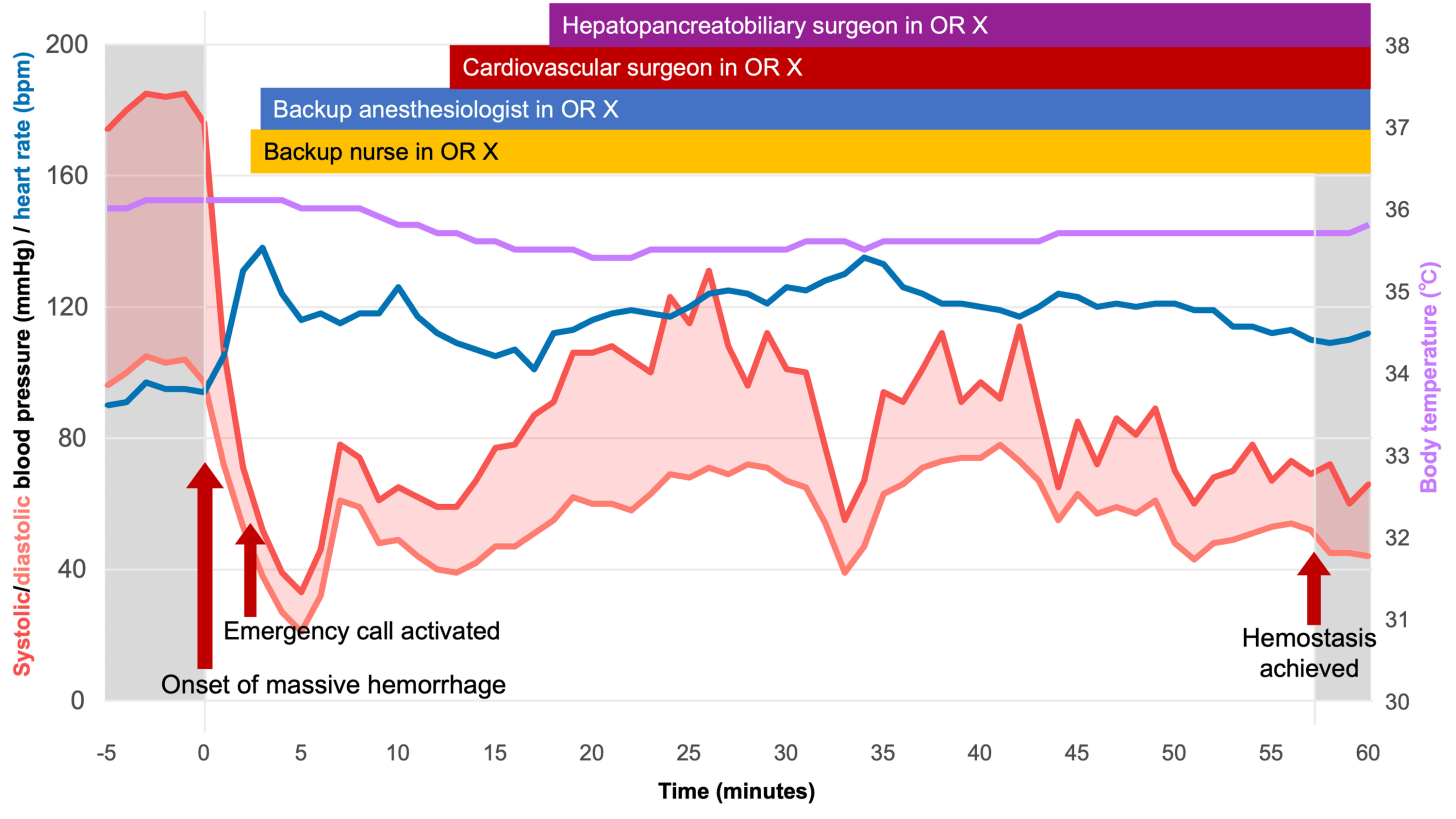
Timeline of physiological changes and multidisciplinary response during the hemorrhagic crisis. Time 0 indicates the onset of hemorrhage. The figure shows temporal changes in vital signs and the timing of key response events, including emergency call activation, transfusion initiation, specialist consultation, and definitive hemostasis. Horizontal bars indicate the arrival of additional personnel, including the backup nurse, backup anesthesiologist, cardiovascular surgeon, and hepatopancreatobiliary surgeon. This figure was created by the authors using R (version 4.5.3; R Foundation for Statistical Computing, Vienna, Austria) and Microsoft PowerPoint. No AI-generated images were used.

Following emergency activation, operating room nurses and clinical engineers performed their assigned tasks in parallel. They prepared transfusion-related equipment, secured the necessary devices, contacted cardiovascular surgeons, and initiated preparation for possible cardiopulmonary bypass support. Blood products were ordered from the blood bank within 1 minute after the arrival of the support team, and transfusion was initiated 10 minutes later. The approximately 10-minute interval between blood product ordering and transfusion initiation was considered to reflect a practical, real-world process time that included emergency release procedures, transport from the blood bank, bedside verification, and initiation of transfusion. The relative contribution of each step could not be separated retrospectively. Cardiovascular surgeons arrived in the operating room 11 minutes after the emergency call, and hepatopancreatobiliary surgeons arrived 16 minutes after the call. The anesthesiologists focused on hemodynamic stabilization; although systolic blood pressure transiently decreased to 30 mmHg, it recovered within approximately 2 minutes. The timeline of physiological changes and multidisciplinary response during the hemorrhagic crisis is summarized in Fig. 2.

After the arrival of the hepatopancreatobiliary surgical team, definitive hemostasis was achieved approximately one hour after hemorrhage onset by ligating the splenic artery proximal and distal to the injured segment. During the hemostatic process, the cardiovascular surgery team proposed aortic balloon occlusion as an additional measure, and the interventional radiology team was urgently mobilized. Based on the interventional radiologist’s assessment that endovascular balloon occlusion was feasible for the injury site, preparation for endovascular hemostasis proceeded in parallel with surgical management. However, because surgical hemostasis was achieved by splenic artery ligation, endovascular hemostasis was ultimately not required. Splenectomy was avoided, and the planned operation was completed.

The total operative time was 10 hours and 16 minutes, and the anesthesia time was 12 hours and three minutes. The total intraoperative blood loss was 4,270 mL. During the intraoperative massive hemorrhage response, the patient received red blood cell concentrate, 2,240 mL; fresh frozen plasma, 1,920 mL; platelet concentrate, 200 mL; cryoprecipitate, 150 mL, prepared from 600 mL of fresh frozen plasma; and isotonic albumin solution, 750 mL. Postoperatively, the patient was transferred to the intensive care unit and managed there for two days. He was discharged on postoperative day 17. The only postoperative complication related to the event was partial splenic infarction, classified as Clavien-Dindo grade I. This infarction was considered attributable to splenic artery ligation; however, partial splenic perfusion was preserved postoperatively.

## Discussion

The principal observation in this technical report is that the SUTO protocol functioned as a shared operational framework during an actual episode of unexpected intraoperative hemorrhage. This clinical deployment was consistent with the core workflow of the SUTO protocol previously described in Japanese [14]. In the present case, emergency activation, declaration of the commander role, transfusion preparation, equipment mobilization, and escalation to additional surgical support were initiated within a short time frame and proceeded in parallel. Interventional radiology support was additionally mobilized during the hemostatic process based on the evolving clinical situation. Although the contribution of the SUTO protocol to the clinical outcome cannot be isolated from surgical, anesthetic, and institutional factors, this clinical deployment illustrates how a locally adapted, role-based response system can support rapid multidisciplinary coordination during a high-acuity operating room crisis. This observation should be interpreted as a process description rather than evidence of clinical effectiveness. The present report demonstrates that the SUTO protocol was activated and that several predefined tasks proceeded in parallel during this clinical deployment; however, it does not establish that the protocol shortened time to transfusion, improved hemostasis, reduced errors, or improved patient outcomes compared with previous practice.

A key feature of the SUTO protocol is that it converts potentially ambiguous tasks into explicitly assigned team actions. During a massive hemorrhage, several critical processes, such as transfusion verification, tracking of blood product availability and use, communication with the blood bank, and preparation of rapid transfusion equipment, may be perceived as tasks that anyone can perform [6,14,16]. However, when responsibility is not clearly assigned, such tasks can paradoxically become delayed or overlooked [16,17]. In the SUTO protocol, these functions are assigned to specific team members, including the nurse managing transfusion, operating room nurses, anesthesiologists, and clinical engineers. This role clarification may allow the team to move from an ad hoc sequential response to a coordinated parallel response, with information regarding blood loss, transfusion preparation, and specialty consultation centralized to the commander.

The case also underscored the importance of building a multilayered hemorrhage-control pathway. In the present case, cardiovascular surgeons, hepatopancreatobiliary surgeons, and the interventional radiology team were mobilized in parallel, maintaining multiple potential strategies for hemorrhage control. The hepatopancreatobiliary surgeons ultimately achieved definitive hemostasis by ligating the splenic artery proximal and distal to the injured segment. At the same time, the cardiovascular surgery team considered aortic balloon occlusion, and the interventional radiology team assessed the feasibility of endovascular balloon occlusion. Although endovascular hemostasis was not ultimately required, maintaining several potential response pathways allowed the team to avoid reliance on a single strategy during a rapidly evolving crisis. This multilayered structure may be particularly important in intraoperative hemorrhage, where the bleeding source, optimal approach, and required expertise may not be immediately apparent.

The present experience also highlights that developing and displaying a protocol is only the first step in institutional preparedness. Operating room teams are not static; staff turnover, rotation of trainees, changes in professional roles, and differences in prior crisis experience can all erode familiarity with emergency workflows over time [10-12]. For this reason, regular simulation should be regarded not merely as an educational activity but as a mechanism for maintaining organizational readiness. Repeated multidisciplinary simulation allows staff members to rehearse role allocation, command structure, communication pathways, transfusion workflow, and escalation procedures under conditions that approximate actual clinical pressure [18].

Simulation and real-world deployment can also reveal weaknesses in the protocol that are not apparent during initial document development [19,20]. In this case, post-event review suggested the need for further refinement, including earlier or simultaneous activation of the interventional radiology team, clearer visual identification of lead nursing roles, and strategies to reduce the cognitive burden placed on the commander and transfusion officer. Following this post-event review, action cards were introduced and are now kept at electronic medical record terminals and beside anesthesia machines; role identifiers and periodic scenario-based drills are similarly employed to prevent task omissions and improve situational awareness [10-12,19]. Thus, the SUTO protocol should be regarded as an iterative operational system that requires repeated testing, feedback, and revision. For ongoing maintenance, daily checks of the massive hemorrhage cart are performed by nursing assistants under the oversight of perianesthesia nurses, and perianesthesia nurses manage the posted cognitive aids. Education for new staff was introduced in 2024, and periodic multidisciplinary simulation was initiated in 2025. Although a fixed formal review interval has not yet been established, protocol revisions are performed as needed based on post-event review, simulation findings, and changes in local operating room workflows.

This report has several limitations. First, it describes a single clinical deployment, and the effect of the SUTO protocol on patient outcomes cannot be evaluated quantitatively. In addition, this report should be interpreted as a descriptive technical report rather than an evaluative implementation study. Although it describes the operational deployment of the SUTO protocol, it was not designed to quantitatively evaluate team performance, time to transfusion, error reduction, or clinical outcomes. Future studies using predefined process measures, before-and-after comparisons, and multicenter validation will be needed. Second, a detailed comparison with cases before protocol implementation was not possible. Third, the workflow depends on institution-specific resources, including blood bank logistics, clinical engineering support, access to cardiovascular and hepatopancreatobiliary surgical support, interventional radiology, and operating room staffing. Fourth, specific time-to-transfusion benchmarks could not be compared against established targets, as no universally accepted threshold for intraoperative massive hemorrhage response has been defined. For these reasons, the SUTO protocol itself may not be directly transferable to all hospitals. Nevertheless, this experience underscores the need to develop local protocols in advance, rather than relying solely on general guidelines, so that emergency workflows reflect each institution’s staffing, resources, communication pathways, equipment availability, and escalation options. The broader process of translating general crisis guidance into a locally adapted, role-based, visible, and simulation-tested operating room response system may be applicable to other institutions.

## Conclusions

The SUTO protocol, an institution-specific response system for intraoperative massive hemorrhage, appeared to provide a shared operational framework for rapid multidisciplinary coordination during an unexpected bleeding crisis. In the reported clinical deployment, transfusion preparation, equipment mobilization, and consultation with additional surgical teams proceeded in parallel within predefined roles and escalation pathways. Interventional radiology support was also mobilized during the hemostatic process as an additional response to the evolving clinical situation. Because crisis-response systems may lose effectiveness as staff and institutional workflows change, regular multidisciplinary simulation and feedback-driven revision are important to maintain organizational readiness.

## References

[1] Smilowitz NR, Ruetzler K, Berger JS (2023). Perioperative bleeding and outcomes after noncardiac surgery. Am Heart J.

[2] Rybaczek M, Kowalski P, Mariak Z, Grabala M, Suszczyńska J, Łysoń T, Grabala P (2025). Safety in spine surgery: risk factors for intraoperative blood loss and management strategies. Life (Basel).

[3] Erdoes G, Faraoni D, Koster A, Steiner ME, Ghadimi K, Levy JH (2023). Perioperative considerations in management of the severely bleeding coagulopathic patient. Anesthesiology.

[4] Kawashima Y, Irita K, Morita K, Tuzaki K, Sawa T (2005). Preoperative hemorrhagic shock and intraoperative bleeding: two main causes of surgical deaths in Japan [Article in Japanese]. J Jpn Soc Blood Transfus.

[5] Irita K, Yoshimura H, Sakaguchi Y, Takamatsu C, Tokuda K (2008). Risk and crisis management by anesthesiologists regarding 'Guidelines for Actions Against Intraoperative Critical Hemorrhage' published by the Japanese Society of Anesthesiologists and the Japan Society of Transfusion Medicine and Cell Therapy [Article in Japanese]. Masui.

[6] Dhoon TQ, Raphael D, Rajan GR, Vaughn D, Engwall S, Vakharia S (2021). Management of massive intraoperative hemorrhage. APSF Newsletter.

[7] Kent J, Thornton M, Fong A, Hall E, Fitzgibbons S, Sava J (2020). Acute provider stress in high stakes medical care: implications for trauma surgeons. J Trauma Acute Care Surg.

[8] Groombridge CJ, Kim Y, Maini A, Smit V, Fitzgerald MC (2019). Stress and decision-making in resuscitation: a systematic review. Resuscitation.

[9] Bijok B, Jaulin F, Picard J (2023). Guidelines on human factors in critical situations. Anaesth Crit Care Pain Med.

[10] Hepner DL, Arriaga AF, Cooper JB (2017). Operating room crisis checklists and emergency manuals. Anesthesiology.

[11] Arriaga AF, Bader AM, Wong JM (2013). Simulation-based trial of surgical-crisis checklists. N Engl J Med.

[12] Agarwala AV, McRichards LK, Rao V, Kurzweil V, Goldhaber-Fiebert SN (2019). Bringing perioperative emergency manuals to your institution: a "how to" from concept to implementation in 10 steps. Jt Comm J Qual Patient Saf.

[13] Jabbour M, Newton AS, Johnson D, Curran JA (2018). Defining barriers and enablers for clinical pathway implementation in complex clinical settings. Implement Sci.

[14] Suto M, Moriyama N, Haruki T, Taniguchi Y, Funaki K, Adachi Y, Otsuki A (2023). Development of an institution-specific massive hemorrhage algorithm: contribution of perianesthesia nurses to patient safety [Article in Japanese]. J Jpn Assoc Oper Room Technol.

[15] Irita K (2014). Present status of critical hemorrhage and its management in the operating room [Article in Japanese]. Rinsho Byori.

[16] Callum JL, Yeh CH, Petrosoniak A (2019). A regional massive hemorrhage protocol developed through a modified Delphi technique. CMAJ Open.

[17] Pettersen G, Gauvin F, Robitaille N, Sansregret A, Lesage S, Levy A (2021). Massive hemorrhage protocol application and teamwork skills. AEM Educ Train.

[18] Redjem ID, Huaulmé A, Jannin P, Michinov E (2025). Crisis management in the operating room: a systematic review of simulation training to develop non-technical skills. Nurse Educ Today.

[19] Long JA, Webster CS, Holliday T, Torrie J, Weller JM (2022). Latent safety threats and countermeasures in the operating theater: a national in situ simulation-based observational study. Simul Healthc.

[20] Weller J, Fahey-Williams K, Henderson K (2025). Resolving latent safety threats identified through in situ simulation: a multicentre mixed-methods study. Adv Simul (Lond).

